# HIV and AIDS, among knowledge, responsibility and ignorance; a study on medical students at the end of their first universitary year

**Published:** 2009

**Authors:** Mircea Ioan Popa, Gabriela Loredana Popa, Anda Mihai, Mădălina Ocneanu, A Diaconu

**Affiliations:** * „Carol Davila” University of Medicine and Pharmacy; ** Romanian Ministry of Public Health, Project Management Unit; *** Colentina Hospital, Bucharest; **** „Bagdasar” Hospital, Bucharest; ***** District Hospital, Sibiu

## Abstract

Despite the global effort to control the spread of HIV and AIDS, the number of HIV-infected people continues to increase worldwide. The failure of present prevention strategies, which rely principally on the modification of behavioral practices that put individuals at risk of getting the infection, and the lack of effective anti-HIV drugs have given an impetus to the search for a better way in the prevention and control of the epidemic.

It was observed and scientifically documented that efforts related to prevention have to be adapted, constantly monitored and readapted in relation to results from periodical surveys. They also have to be well sustained over a sufficiently long period of time (years).

Under these assumptions, in order to evaluate the knowledge and perception/attitudes of the students in the University of Medicine, we developed a questionnaire that we presented for completion, for example, to medical students at the end of their first year of study.

The gathered data conducted us to the assumption that information regarding HIV transmission is not yet internalized in order to determine specific practices, and attitudes are more related to perceptions and beliefs than to facts. An active, responsible, adult day to day responsibility could increase the protection against HIV transmission.

A limitation of our study was represented by the reduced number of participants (96) and the lack of correlation between attitudes and knowledge, between students in different years of study and over time. It is our intention to continue this research and provide more complete data in the nearest future.

## Background

Despite the global effort to control the spread of HIV and AIDS, the number of HIV-infected people continues to increase worldwide. The failure of present prevention strategies, which rely principally on the modification of behavioral practices that put individuals at risk of getting infected, and the lack of effective anti-HIV drugs have given an impetus to the search for a vaccine against HIV. HIV, the causative agent of AIDS, has infected more than 47 million people worldwide, since the start of the epidemic over 25 years ago, with 3.5 to 6.6 million of new infections in 2005 (UNAIDS report). The death toll has reached 14 million adults and children with an estimated 2.5 to 3.5 million of those deaths occurring during 2006 (UNAIDS). [**1**-**3**] The precise numbers of people living with HIV, people who have been newly infected or who have died with AIDS are not known. Current HIV prevention activities focus on identifying and modifying behavioral practices that put individuals at risk of acquiring a HIV infection. [**4**-**8**] These activities are very important, but will probably not be enough to curb the global epidemic. On the other hand, it is outstanding to have information regarding attitude, knowledge and responsibility of medical staff, starting with the future medical doctors.

Only after 1985 the problems related to HIV infection, and AIDS, started to be discussed also in Romania. Although so many years have passed, despite huge financial efforts, worldwide, neither curative treatment nor preventive vaccine were identified and established to date. Most of the efforts with limited, but visible effects/results are oriented towards prevention through increase of knowledge related to HIV transmission in order to enable population and medical professionals to limit the spread of the infection. It was observed and scientifically documented that efforts related to prevention have to be adapted, currently monitored and readapted in relation to results from periodical surveys and, maybe most important, to be well sustained over a sufficiently long period of time (years).

## Material and methods

Under these premises, in order to evaluate the knowledge and perception/attitudes of the students in the University of Medicine we developed a questionnaire that we presented for completion, for example, to medical students at the end of their first year of study. The questionnaire was multiplied and each student received a personal list of questions and 1 page in order to complete the answers. The questionnaire was self-administered, after a short presentation of the study, objectives, and terms of completion. The answers were introduced in a data base to be analyzed. The answers of the sample (96 students) will be presented.

## Results

The first part of the questionnaire included demographic data regarding students who accepted to participate to the study. Thus the majority of the questioned students were females (65.7%) and only 5 students (5.2%) were married, from the total of 96 registered forms. 

Personal history regarding HIV testing of the young medicine students showed that a small number of students have requested a test to identify the presence of HIV antibodies (2,63% more than once, and 13,15% once, respectively).

We can observe a significant association between personal fear regarding the HIV/AIDS infection and, the perception of personal HIV infection risk, the answers being presented in **Table no. 1** and **Table no. 2**. Even regarding personal fear related to HIV infection the highest proportion of respondents announced that is “not so much” (40.6%), personal risk perception was mentioned as “high” for more than one third of the respondents (32.29%). 

**Table 1:** 

Personal fear	
Not so much	40,62%
Small	28,12%
High	31,26%

**Table 2:** 

Personal risk perception	
High	32,29%
Medium	13,44%
Low	13,44%
Very low	16,66%
None	24,17%

From the total sample only 11 (11.46%) students completing the questionnaire personally knew a positive HIV infected person at the time of the investigation. That percentage can be correlated with the approximately high percentage of respondents who considered themselves not being at risk at all (24.17%).

Regarding HIV transmission modalities, the participating students answered that HIV can be transmitted through:

- Sexual intercourse: 90.62%

- Contact with/from public toilet: 13.44%

- Sharing the mechanical shaving machine with a person: 60.42%

- A dental treatment: 78.13%

- Sharing a glass with an infected person: 14.58%

- Sharing the same hospital room with an HIV infected person: 6.25%

- Sharing intravenous drug injections: 62.5%

- Contact with contaminated saliva: 23.96%

- Blood transfusion: 82.29%

- Blood donation: 54.17%

- An acupuncture session: 44.79%

- A mosquito sting: 14.58%

Even most of the students identified the principal ways for HIV transmission (sexual intercourse, blood transfusion, sharing intravenous drug injections), we can remark that most of them (a large proportion) had also, incorrect knowledge/information, considering that the virus can be transmitted by using the public toilet, sharing a glass, donating blood etc. (in a proportion between 6.25% and 54.17%).

The data regarding student attitudes toward an HIV infected person are presented in **Table no. 3**. 

**Table 3:** 

They will/can continue to see that person	65.63%
They will/can go on holidays, together	52.08%
They will/can work in the same place	72.92%
They will leave their kids with that person	13.44%
They will have meals, together	55.21%

If we consider proportion of misinformation regarding HIV transmission in relation with attitudes towards an HIV infected person we cannot identify a specific correlation: positive and non-discriminatory attitudes prevail in large proportion (with the exception of their kids in relation with HIV infected persons). That can conduct us to the assumption that information regarding HIV transmission is not yet internalized in order to determine specific practices and attitudes. Moreover, attitudes are more in relation with perceptions and beliefs than facts (ex. The need for own child protection modifies all the attitudes related to the possibility of infection). We can continue with a further assumption that an active, responsible, adult day to day responsibility can increase the protection against HIV transmission.

In order to determine the degree of acceptance of HIV persons in society, students were asked about possibly/compulsory measures to prevent HIV transmission. As far as measures to be taken are considered, the students responded:

- Forbidding school access to AIDS children: 20.83%

- Considering legitimate to exclude persons with AIDS from work places: 20.83%

- Considering measures aimed at isolating the infected persons: 16.66%

- Considering compulsory ELISA testing without the informed consent: 55.21%

- Considering legitimate homosexuality punishment: 36.46%

- Considering compulsory announcement of HIV status at the workplaces: 65.63%.

Responses to all items related to social acceptance of HIV/AIDS individuals showed again, that the respondents disregarded the correct information related to HIV transmission in their answers in a proportion higher than almost 20%, to 65%. This conclusion underlines the issues mentioned before regarding the relation between information, attitudes and practices. 

Also, we can observe a discrepancy between apparently more tolerant attitudes announced in the previous group of questions regarding personal attitudes and this one regarding social and legal measures to be taken in order to protect general public health and to restrain HIV transmission (as if each individual is not part of the society). These determined us to think that there is a kind of understanding of the necessity/recommendation to be tolerant, as information received through different channels, but, again, not transformed in a consistent attitude and practice. 

**Table no. 4** includes the percentage of respondents’ trust regarding reliability on different structures in providing correct and relevant data. The percentage represents institutions and persons/professionals that students did not consider that are providing true and relevant information related data about HIV/AIDS infection.

**Table 4:** 

HIV/AIDS prevention organizations	6,25%
Health Ministry	21,88%
Researchers	8,33%
Government	67,71%
Doctors	8,33%
Journalists	72,92%
Medical Professors	6,25%
Local elected persons	69,73%

The data collected shows a high level of confidence in scientifically and qualified professionals and institutions: medical professors, doctors, researchers, HIV/AIDS prevention organizations.

A very low level of confidence is attributed to central and local authorities. That can lead to different conclusions: a possible national trend related to these data and the fact that correct and complete information related to HIV/AIDS infection can be hardly obtained (in the view of the majority of respondents) due to the fact that trustful sources are considered only specific professionals.

Regarding the impact of mass media campaigns upon respondents, students declared that these made them:

- Feel closer to the affected persons: 34.37%

- Be concerned regarding a personal risk: 67.70% and, respectively regarding the risk for the society: 76.04%

- Use condoms: 34.37%

- Have less sexual partners: 29.16%

- Have an HIV test (ELISA): 27.08%

From the results of the above group of answers it seems that mass media campaigns have a higher impact on emotional aspect of individuals questioned regarding infected persons, respectively personal and more social risk (empathy, concern) than they have on changing personal behaviors (using condoms, number of sexual partners, HIV testing). If we put in relation the answers regarding reliability that those respondents have on HIV/AIDS prevention organizations (93.75%), we will notice that information from trustful sources, transmitted even through media campaigns is not enough to determine significant changes in medical students’ behavior related to HIV prevention. 

The students investigated agreed to the following affirmations:

- Condoms kill romance: 46,87%

- Condoms diminish sexual pleasure: 48,95%

- Would buy condoms if they were cheaper: 11,45%

- When you love somebody you do not need a condom: 6,25%

- Condom use would diminish partners’ trust in each other: 11,45%

- Condoms are reserved only for young people: 6,25%

- Condoms are difficult to use: 5,20%

- It is shameful to buy a condom: 5,20%

- Total agreement about the fact that mass media should try to explain condom use: 51,04%

- Condoms are totally old fashioned: 2,08%

From the proportion of these affirmations’ result most of the “older” barriers in condom use and myths have been overcome (price, negative influence of condom use in trust and love, condom use in relation with shame, young age, and difficulty in use). Anyhow, the most frequent announced barrier in condom use, regarding the impact on sexual pleasure and spontaneity is claimed by almost half of the respondents. In addition, a third part of the respondents declared that personal fear and personal risk for HIV infection is high.

**Table no. 5** presents students’ considerations regarding HIV/AIDS infection in our country, the data being gathered untill 1996.

**Table 5:** 

HIV/AIDS infection is a very serious problem	56,25%
Mass media treated this subject with great responsibility	15,62%
Ministry of Health treated this subject with great responsibility	11,45%
During the last years, all the efforts were concentrated so as to limit the spreading of the infection	8,33%
Wish to receive more information regarding HIV/AIDS infection	7,70%
Wish to participate in HIV/AIDS prevention campaigns	40,62%

It can be seen that the youth’s attitude regarding AIDS has changed over the years especially due to increased access to more and accurate information and intensive prevention interventions. Data presented above can be used as a start/baseline for the future observing trends/developments regarding knowledge, attitudes and practices of these group of young future doctors, and also to establish an hierarchy of priorities in developing further prevention campaigns, advocacy intervention and more directive evaluations and interventions.

## Discussions and conclusions

Research from developed countries has just as much to contribute to the global fight against AIDS as the one from rich countries. In many ways, it is even more relevant to these countries with the vast majority of the world's HIV infections.

In our study, even most of the students identified the main ways for HIV transmission. We remarked that quite a large proportion had also, incorrect knowledge/information, taking into account the fact that the virus can be transmitted by using public toilet, sharing a glass, donating blood, etc.

The data gathered conducted us to the assumption that information regarding HIV transmission is not yet internalized in order to determine specific practices and attitudes and attitudes are more in relation with perceptions and beliefs than with facts. An active, responsible, day to day, adult responsibility could increase the protection against HIV transmission.

Moreover, we observed a discrepancy between apparent more tolerant attitudes mentioned in one group of questions regarding personal attitudes and the others regarding social and legal measures to be taken in order to protect general public health and to restrain HIV transmission. These determined us to think that there is a kind of understanding of the recommendation to be tolerant, as information received through different channels, but, again, not transformed in a consistent attitude and practice. 

The data collected showed a high level of confidence in scientists and qualified professionals and institutions: medical professors, doctors, researchers, HIV/AIDS prevention organizations.

On the other hand, a very low level of confidence was attributed to central and local authorities. That can conduct to different conclusions: a possible national trend related to these data and the fact that correct and complete information related to HIV/AIDS infection can be obtained (in the view of the majority of respondents) with difficulty due to the fact that trustful sources are considered only specific professionals. 

The present study has shown that there is insufficient and inaccurate information regarding HIV/AIDS on different channels and we suggest that more attention should be paid to the development of adequate AIDS websites in our country together with supplementary information transmitted to the future medical doctors in the faculty.

A limitation of our study was the number of the participants and the lack of correlation between attitudes and knowledge, between students in different years of study and over time. It is our intention to continue this research and provide more complete data in the nearest future. 

**Acknowledgements**

The authors would like to thank to Dr. Dana Podaru, GF Project Expert, for her thorough and insightful comments and review on this document. 
